# The effect of climatic factors on the number of malaria cases in an inland and a coastal setting from 2011 to 2017 in the equatorial rain forest of Cameroon

**DOI:** 10.1186/s12879-022-07445-9

**Published:** 2022-05-13

**Authors:** Raymond Babila Nyasa, Fuanyi Awatboh, Tebit Emmanuel Kwenti, Vincent P. K. Titanji, Ndip Lucy M. Ayamba

**Affiliations:** 1 Department of Microbiology and Parasitology, University of Buea, P. O. Box 63, Buea, Cameroon; 2 Biotechnology Unit, Faculty of Science, University of Buea, P.O. Box 63, Buea, South West Region Cameroon; 3 Department of Medical Laboratory Sciences, Faculty of Health Science, University of Buea, P.O. Box 63, Buea, South West Region Cameroon; 4 Cameroon Christian University, P.O. Box 5, Bali, North West Region Cameroon; 5 Laboratory for Emerging Infectious Diseases, University of Buea, P.O.Box 63, Buea, South West Region Cameroon

**Keywords:** Malaria, Humidity, Temperature, Rainfall, Climate, Titko, Muyuka, Cameroon, Climate

## Abstract

**Background:**

Weather fluctuation affects the incidence of malaria through a network of causuative pathays. Globally, human activities have ultered weather conditions over time, and consequently the number of malaria cases. This study aimed at determining the influence of humidity, temperature and rainfall on malaria incidence in an inland (Muyuka) and a coastal (Tiko) settings for a period of seven years (2011–2017) as well as predict the number of malaria cases two years after (2018 and 2019).

**Methods:**

Malaria data for Muyuka Health District (MHD) and Tiko Health District (THD) were obtained from the Regional Delegation of Public Health and Tiko District Health service respectively. Climate data for MHD was obtained from the Regional Delegation of Transport while that of THD was gotten from Cameroon Development Coorporation. Spearman rank correlation was used to investigate the relationship between number of malaria cases and the weather variables and the simple seasonal model was used to forecast the number of malaria cases for 2018 and 2019.

**Results:**

The mean monthly rainfall, temperature and relative humidity for MHD were 200.38 mm, 27.05^0^C, 82.35% and THD were 207.36 mm, 27.57 °C and 84.32% respectively, with a total number of malaria cases of 56,745 and 40,160. In MHD, mean yearly humidity strongly correlated negatively with number of malaria cases (r = − 0.811, p = 0.027) but in THD, a moderate negative yearly correlation was observed (r = − 0.595, p = 0.159). In THD, the mean seasonal temperature moderately correlated (r = 0.599, p = 0.024) positively with the number of malaria cases, whereas MHD had a very weak negative correlation (r = − 0.174, p = 0.551). Likewise mean seasonal rainfall in THD moderately correlated (r = − 0.559, p = 0.038) negatively with malaria cases, contrary to MHD which showed a very weak positive correlation (r = 0.425, p = 0.130). The simple seasonal model predicted 6,842 malaria cases in Muyuka, for 2018 and same number for 2019, while 3167 cases were observed in 2018 and 2848 in 2019. Also 6,738 cases of malaria were predicted for MHD in 2018 likewise 2019, but 7327 cases were observed in 2018 and 21,735 cases in 2019.

**Conclusion:**

Humidity is the principal climatic variable that negatively influences malaria cases in MHD, while higher seasonal temperatures and lower seasonal rain fall significantly increase malaria cases in THD.

**Supplementary Information:**

The online version contains supplementary material available at 10.1186/s12879-022-07445-9.

## Background

Malaria is the leading endemic, parasitic, infectious disease in Africa as a whole and in Sub-Saharan Africa in particular. Children under 5 years and pregnant women are at the highest risk of severe malaria. In 2019 an estimated 229 million cases of malaria occurred in 87 countries and 409,000 deaths were recorded [1]. Cameroon is amongst the 11 countries that accounted for 70% of the global burden of malaria and 71% of the global estimated deaths from malaria [1]. In 2017, Cameroon alone accounted for 16% of the 45 million estimated cases of malaria in Sub-Saharan Africa [2]. There was no difference in the case incidence of malaria, in 2020 compared to 2015 in Cameroon [1]. In Cameroon malaria accounts for about 48% of all hospital admissions and 30% of all hospital deaths [3].

Programs have been put in place to prevent malaria as well as to decrease their transmission. Such programs have recorded success in the control of malaria but despite these successes, malaria remains the principal infection in Africa [4]. Studies have shown that temperatures of 18-320C and a humidity of at least 60% are optimal for Anopheles mosquitoes to survive long enough to acquire and transmit the malaria parasite [5, 6]. Again, in the range of 18-260C, an increase of 10C in temperature changes the lifespan of a mosquito by more than a week [7]. An increase in rainfall leads to an increase in the amount of standing water which provides breeding ground for mosquitoes [8] but heavy rains washes off breeding sites [9] thereby reducing mosquito population and consequently malaria transmission. It has also been reported that at humidity lower than 60% the life span of mosquitoes decreases and transmission does not occur [10]. These therefore show that weather fluctuations have an impact on the number malaria cases.

It has been reported that human activities especially burning of fossil fuels and deforestation leads to fluctuation in weather and climatic conditions [11]. It is also known that the climate of a coastal and an inland setting does not change at the same rate. The coastal settings have a more moderate weather conditions than the inland settings. This is because the land heats up faster during the day and loses the heat quickly during the night in an inland setting, leading to extremes of temperature and relative humidity, while in the coastal settings, the sea or ocean heats up and cools down more slowly thereby keeping the temperature and relative humidity approximately at the same levels [12]. Likewise coastal areas regularly suffer from convectional rainfall and are usually more humid than inland areas. Considering a coastal setting (Tiko Health District) and an inland setting (Muyuka Health District), all of which are town in the South West Region of Cameroon, this study seeks to find out how weather fluctuations have been affecting the number of malaria cases in the two towns for a seven-year period (2011 to 2017) and forecasting future number of cases for a two-year period using the simple seasonal model.

## Methods

### Study site

**Fig. 1:**
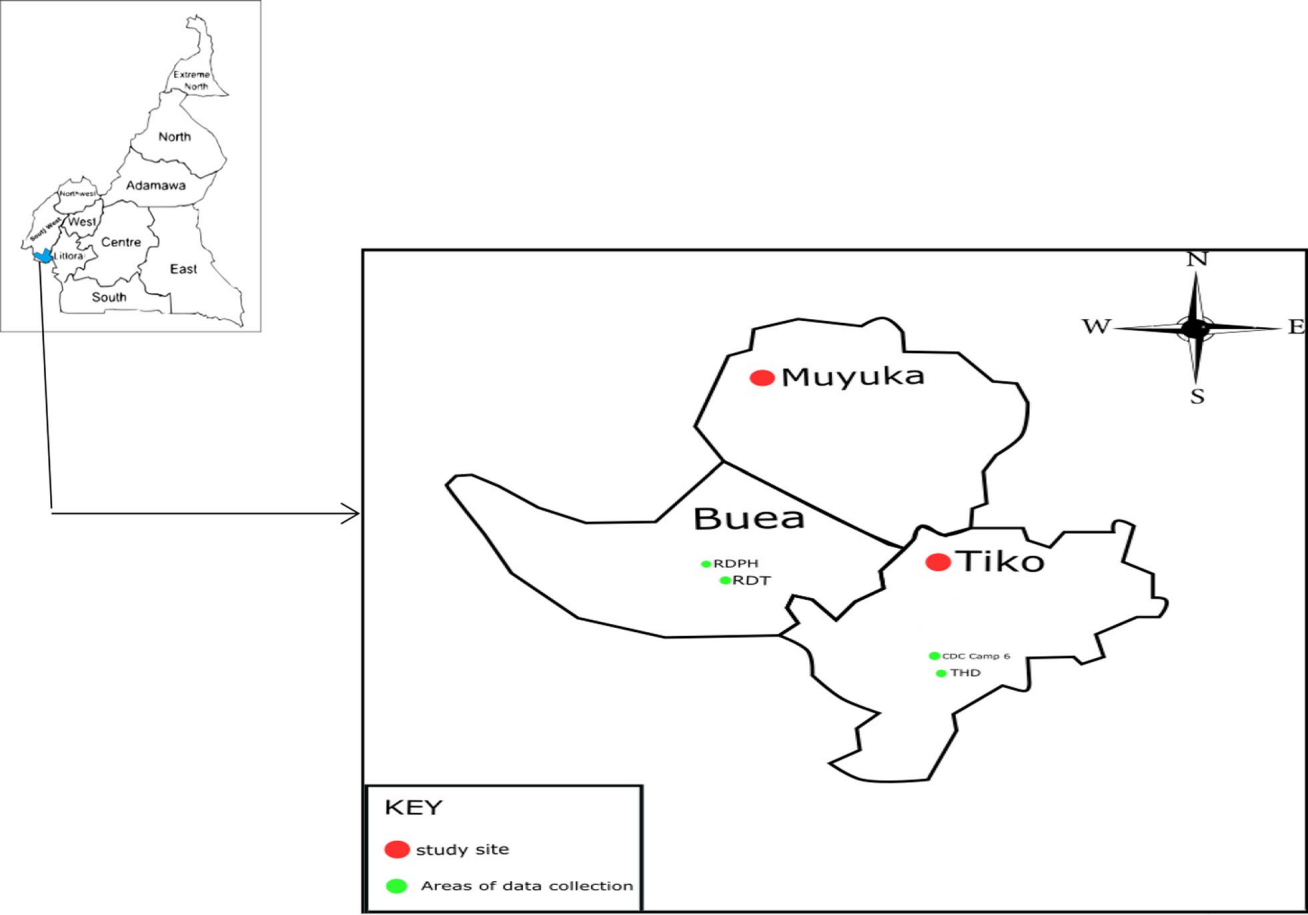
Map of study sites (drawn using MapInfo Professional 11.5 software and edited with Inkscape software)

The study was carried out in Tiko Health District (THD) and Muyuka Health District (MHD). Tiko is a coastal town in Fako division of the South West Region of Cameroon (Fig. 1). It is located between latitude 9.5338889 N to 9.6691667 N and longitudes 9.4186111E to 9.9186111E [13], and it is situated at an elevation of 64 m above sea level with a population of 55,914 as at 2010. Tiko District Health Service is where all information concerning health in Tiko is stored. Tiko District Health Services has 7 health areas and about 17 health facilities under its control. The average weather in Tiko is warm with temperature ranging from 23 °C to 32 °C and relative humidity ranging from 59 to 100% [14]. Tiko Health District has an annual rainfall of 2088 mm with the rainy season running from March to October and the dry season from November to February [15]. The Cameroon Development Corporation is the largest Agro-Industrial Complex in Tiko, that grows, processes and markets tropical export crops. Its major products include banana, semi-finished rubber, palm oil and palm kernel oil. The company has been existing since 1947 till date and it has established weather stations throughout Tiko, from which it uses climatic data to trouble shoot causes of low yield, when they do occur.

Muyuka is an inland town in Fako Division of Cameroon, with a population of 34,296 in 2010, where this study was carried out (Fig. 1). It is located between latitude 4.27524 N to 4.31222 N and longitude 9.40104E to 9.42945E and situated at an elevation of 305 m above sea level. Muyuka is found on the leeward side of Mount Fako with an average temperature ranging from 27.5 to 280C in the month of February [16]. Muyuka has a rainfall of of less than 1612 mm on an average every year [16] with the rainy season running from March to October and the Dry season from November to February.

### Study design and data collection

It was a retrospective study design involving the review of records to investigate the relationship between weather variations and the number of malaria cases in the study area from 2011 to 2017. Climatic data obtained for this study were average monthly historical rainfall, temperature and relative humidity records in Tiko and Muyuka Health Districts obtained from the Cameroon Development Corporation Tiko Camp7 Group Banana plantation office and the Regional Delegation of Transport respectively. From this data, average yearly data on each climatic variable was calculated from the average of the months that make up the year, while average seasonal data on each climatic variable was calculated by taking the average of the dry season months (November to February) and that of the rainy season months (March to October). Data on the population and monthly number of malaria cases in Tiko, was from the Tiko District Health Service obtained through their online District Health Information System 2 (DHIS2) portal and that of Muyuka Health District was obtained from the Regional Delegation of Public Health (RDPH) for the period of January 2011 to December 2017. The yearly incidence of malaria was gotten from the total number of malaria cases in each year. In Cameroon, routine diagnosis of malaria is by rapid diagnostic test (RDT), performed according to the manufacturer’s instruction and the gold standard microscopy is used more for confirmatory diagnosis; which involves staining of thick blood film made from finger prick, with 10% Giemsa solution for 20 min and observing the air-dried slide under oil immersion (X 1000) magnification of the light microscope for the presence of malaria parasites.

### Ethical considerations

Administrative authorization for this study was gotten from the Regional Delegation of Public Health reference number 1211/MINSANTE/SWR/RDPH/PS/959/741 and an ethical clearance was obtained from the University of Buea Faculty of health Sciences-Institutional Review Board (UBIRB) reference number 2019/996–06/UB/SG/IRB/FHS.

### Data analysis

All data were entered into Microsoft Excel. The analysis of the data was done using SPSS Statistics for Windows version 25.0 (SPSS Inc., Chicago, II, USA). The incidence of malaria for the different years was gotten as the total number of malaria cases for that year. The monthly, yearly, and seasonal averages of climatic variables (rainfall, temperature and humidity) were calculated with Microsoft Excel 2019. The charts and plots were produced using Qtiplot. The correlation between weather parameters; temperature, humidity and rainfall in Muyuka Health District (MHD) and Tiko Health District and the monthly, seasonally and yearly number of malaria cases were performed by Spearman’s rank correlation. Also, the simple seasonal model in SPSS Statistics for Windows version 25.0 (SPSS Inc., Chicago, II, USA), which is applied on series with no trend and a simple seasonal effect that is constant over time, was used to predict the number of malaria cases for a two-year period (2018 and 2019), which also showed the stationary R-squared and ljung box statistics.

## Results

### Malaria incidence in an inland (Muyuka) and a coastal (Tiko) setting

**Fig. 2:**
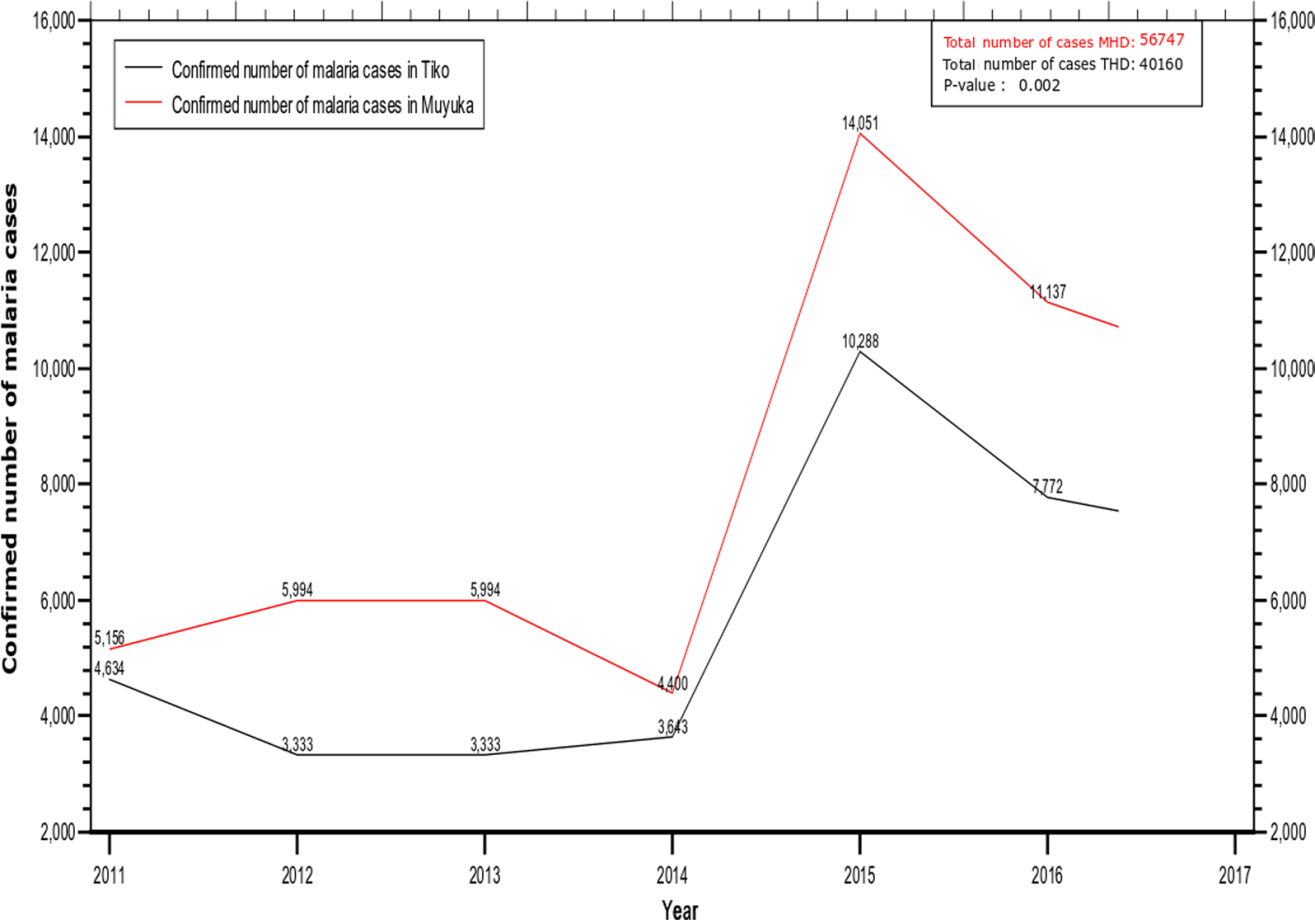
Trend of malaria prevalence in Muyuka and Tiko Health districts from 2011 to 2017

The incidence of malaria in Muyuka and Tiko Health Districts was defined as the number of malaria cases reported by the hospitals each year. An overview of malaria incidence in Muyuka and Tiko shows that more cases of malaria were reported in Muyuka (56,747) than in Tiko (40,160) for the seven years under consideration as shown in Fig. 2 (p = 0.018). The trend in malaria incidence between Muyuka and Tiko is different from 2011 to 2014 but follows the same pattern from 2014 to 2017. The lowest incidence in Muyuka was observed in the year 2014 (4400) while the lowest incidence in Tiko was observed in 2012 (3333) and 2013 (3333). In Muyuka, the incidence of malaria increased from 5156 in 2011 to 5994 in 2012 as opposed to Tiko where the incidence reduced from 4634 in 2011 to 3333 in 2012. Both settings had a constant incidence from the 2012 to 2013 from of 5994 in Muyuka and 3333 in Tiko. From 2015 to 2017 the incidence of malaria saw a steady decline in Tiko from 10,288 in 2015 to 7772 in 2016 and finally 7157 in 2017. Also, the incidence of malaria decreased from 2015 to 2017 in Muyuka from 14,051 in 2015 to 11,137 in 2016 and finally to 10,015 in 2017. MHD had a mean monthly rainfall of 200.38 mm, temperature of 27.05 °C and relative humidity of 82.35% while THD had mean values of 207.36 mm, 27.57 °C and 84.32% respectively, from 2011 to 2017.

### Influence of humidity on the number of malaria cases in Muyuka and Tiko Health Districts from 2011 to 2017

**Fig. 3:**
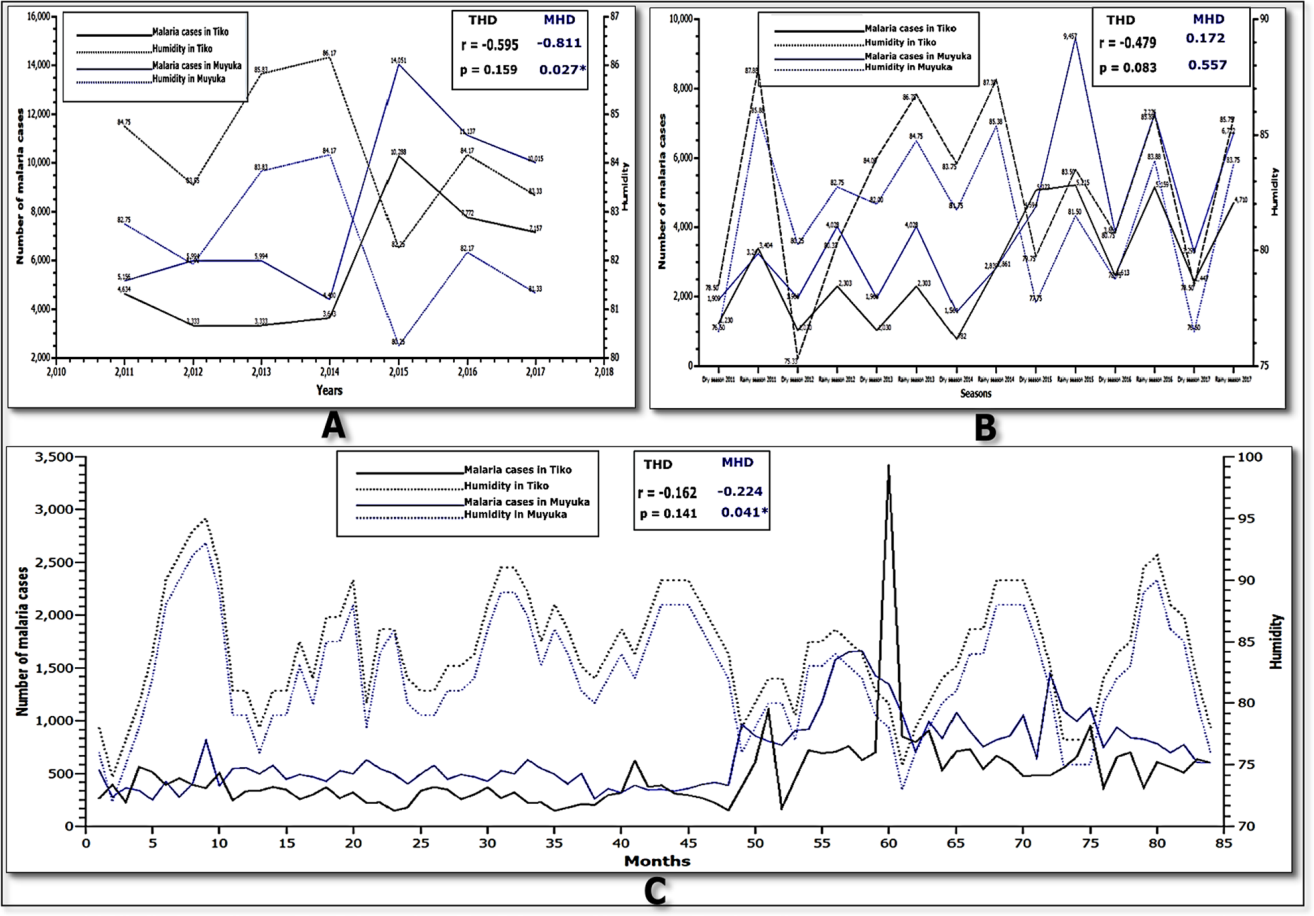
Relationship between yearly (**A**), seasonal **B** and monthly **C** humidity with number of malaria cases in Muyuka and Tiko Health Districts respectively, from 2011 to 2017

The correlation of humidity and the number of malaria cases was calculated; yearly (Fig. 3A), seasonally (Fig. 3B) and monthly (Fig. 3C) for Tiko and Muyuka health districts. The yearly and monthly correlation of humidity with number of malaria cases in MHD showed significantly strong and very weak negative correlation (r = − 0.811, and p = 0.027) and (r = − 0.224, and p = 0.041) respectively, although seasonal data of humidity did not show any significant correlation with number of malaria cases (r = 0.172 and p = 0.557). In THD there was a moderate, weak and very weak negative correlation between yearly (r = -0.595 and p = 0.159), seasonally (r = − 0.479 and p = 0.083) and monthly (r = − 0.162 and p = 0.141) humidity respectively, with number of malaria cases, which were not significant.

### Influence of temperature on the number of malaria cases in Muyuka and Tiko Health Districts from 2011 to 2017

**Fig. 4:**
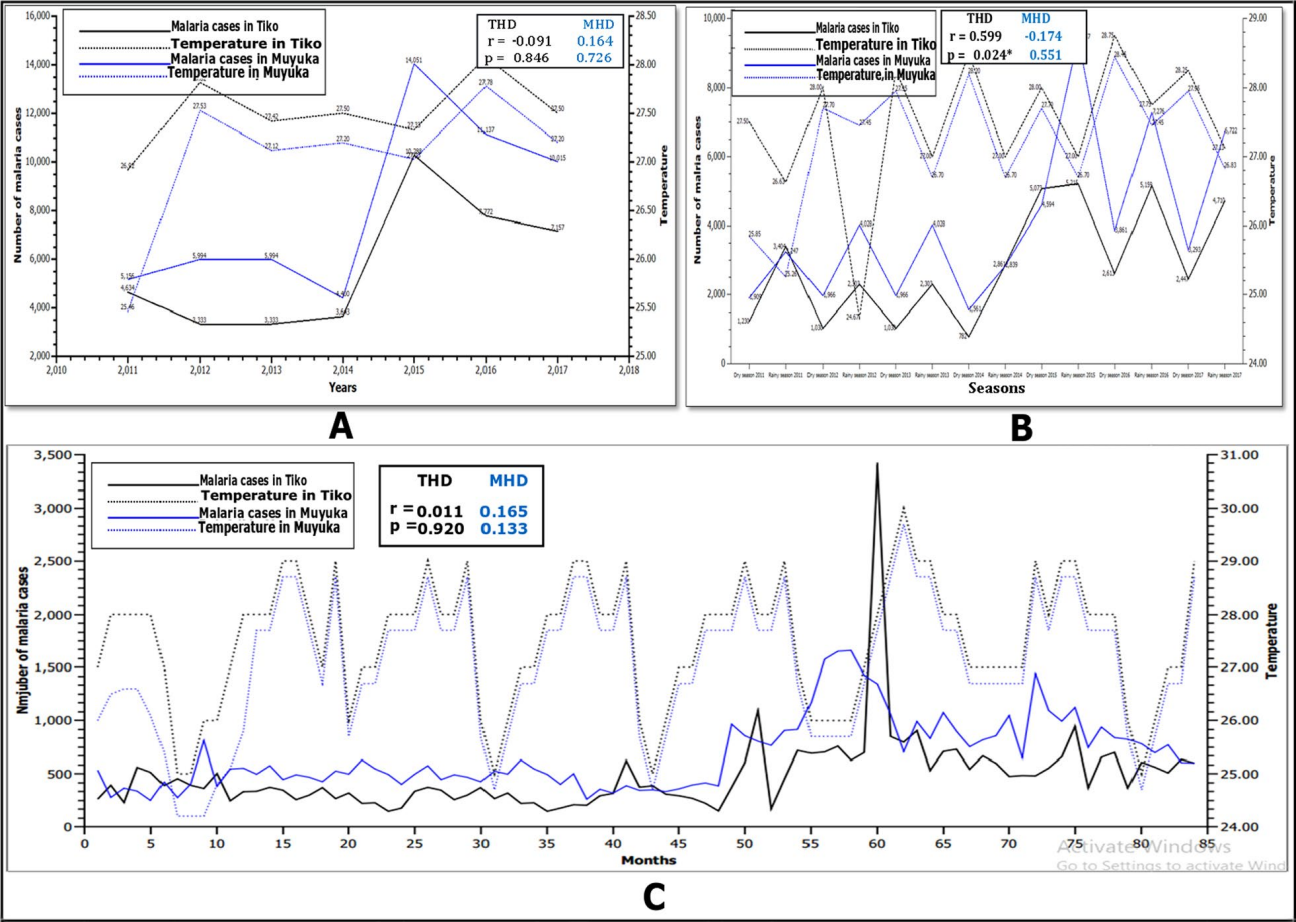
Fig. 1 Relationship between yearly (**A**), seasonal **B** and monthly **C** temperatures with number of malaria cases in Muyuka and Tiko Health Districts respectively from 2011 to 2017

On a yearly and monthly basis, average temperature correlated very weak positively with number of malaria cases in MHD (r = 0.164 and p = 0.726), and (r = 0.165 and p = 0.133) respectively, although average seasonal temperature correlated very weakly negatively with number of malaria cases (r = − 0.174 and p = 0.551), but these correlations were not significant (Fig. 4A–B). Seasonal correlation of average temperature with number of malaria cases in THD showed a moderately positive correlation (r = 0.599 and p = 0.024). While yearly average temperature showed a very weak negative correlation (r = − 0.091 and p = 0.846) with number of malaria cases and monthly average temperature also showed a very weak positive correlation (r = 0.011 and p = 0.920) with the number of malaria cases.

### Influence of rainfall on the number of malaria cases in Muyuka and Tiko Health Districts from 2011 to 2017

**Fig. 5:**
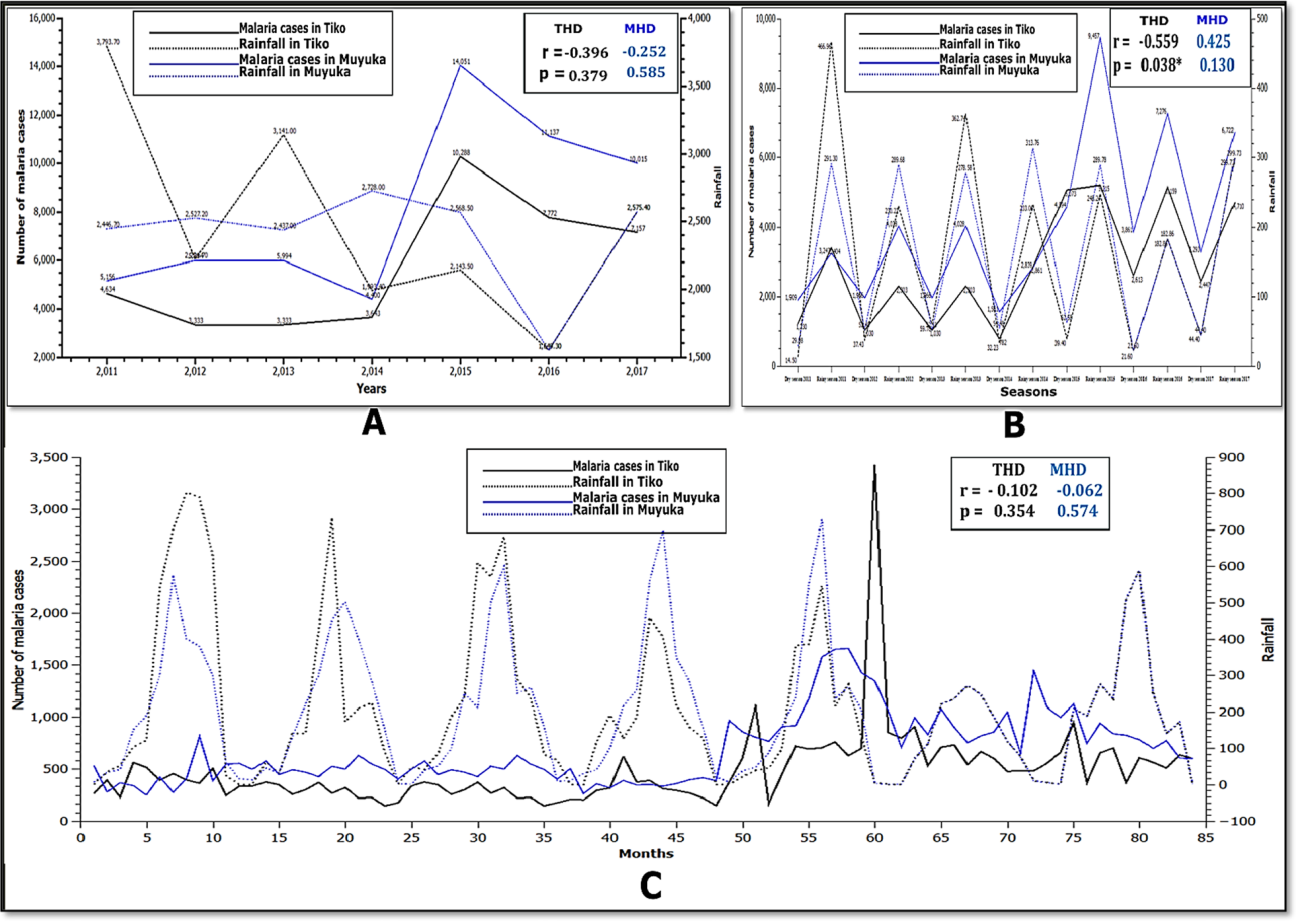
Fig. 1 Relationship between yearly (**A**), seasonal **B** and monthly **C** rainfall with number of malaria cases in Muyuka and Tiko Health Districts respectively, from 2011 to 2017

Average yearly and monthly rainfall in MHD showed very weak negative correlations with number of malaria cases (r = − 0.252 and p = 0.585) and (r = − 0.062 and p = 0.574) respectively (Fig. 5A and C), but there was a weak positive correlation with mean seasonal rainfall (r = 0.425 and p = 0.130) (Fig. 5B). On a whole, yearly and monthly average rainfall in THD showed a weak (r = − 0.396 and p = 0.379) and very weak (r = − 0.102 and p = 0.354) negative correlation with number of malaria cases, respectively. While average seasonal rainfall showed a moderately negative correlation with number of malaria cases (r = − 0.559 and p = 0.038) in THD.

### Trend of malaria cases in Muyuka and Tiko Health Districts from 2011 to 2017

**Fig. 6:**
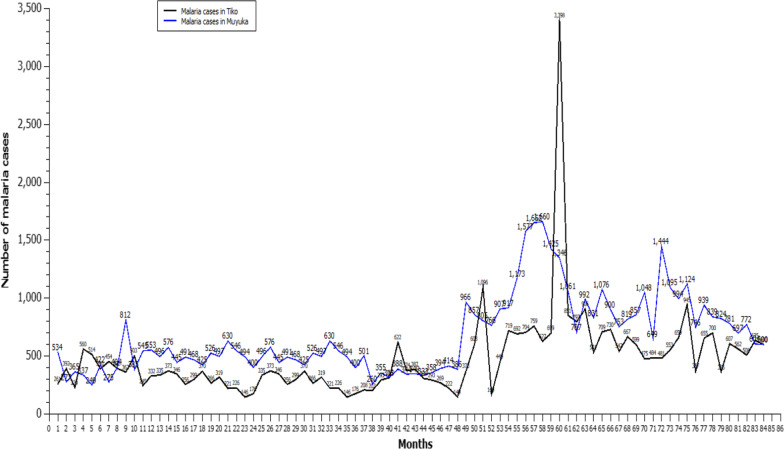
Monthly trends of malaria cases from 2011 to 2017 in Muyuka and Tiko Health District (1 = January 2011 to 84 = December 2017)

Trend in the number of malaria cases in Muyuka and Tiko Health Districts showed some similarities and differences (Fig. 6). In both settings, there was an unusually high number of malaria cases in 2015 with the highest number recorded in October (1660) in Muyuka and 3398 in Tiko in December. Generally, the number of malaria cases in Tiko follows a quadratic trend (the values of a time series tend to rise and fall at a rate that is not constant; it changes over time), therefore second differencing is required to make the trend stationary. In Muyuka the trend is rather linear on a general basis. This is seen because there is an increased number of malaria cases from 2011 to 2017 except for 2014 and part of 2017 which can be represented by a straight line as such, first differencing is required to make the trend stationary.

### Prediction of number of malaria cases in 2018 and 2019 using the simple seasonal model

**Fig. 7:**
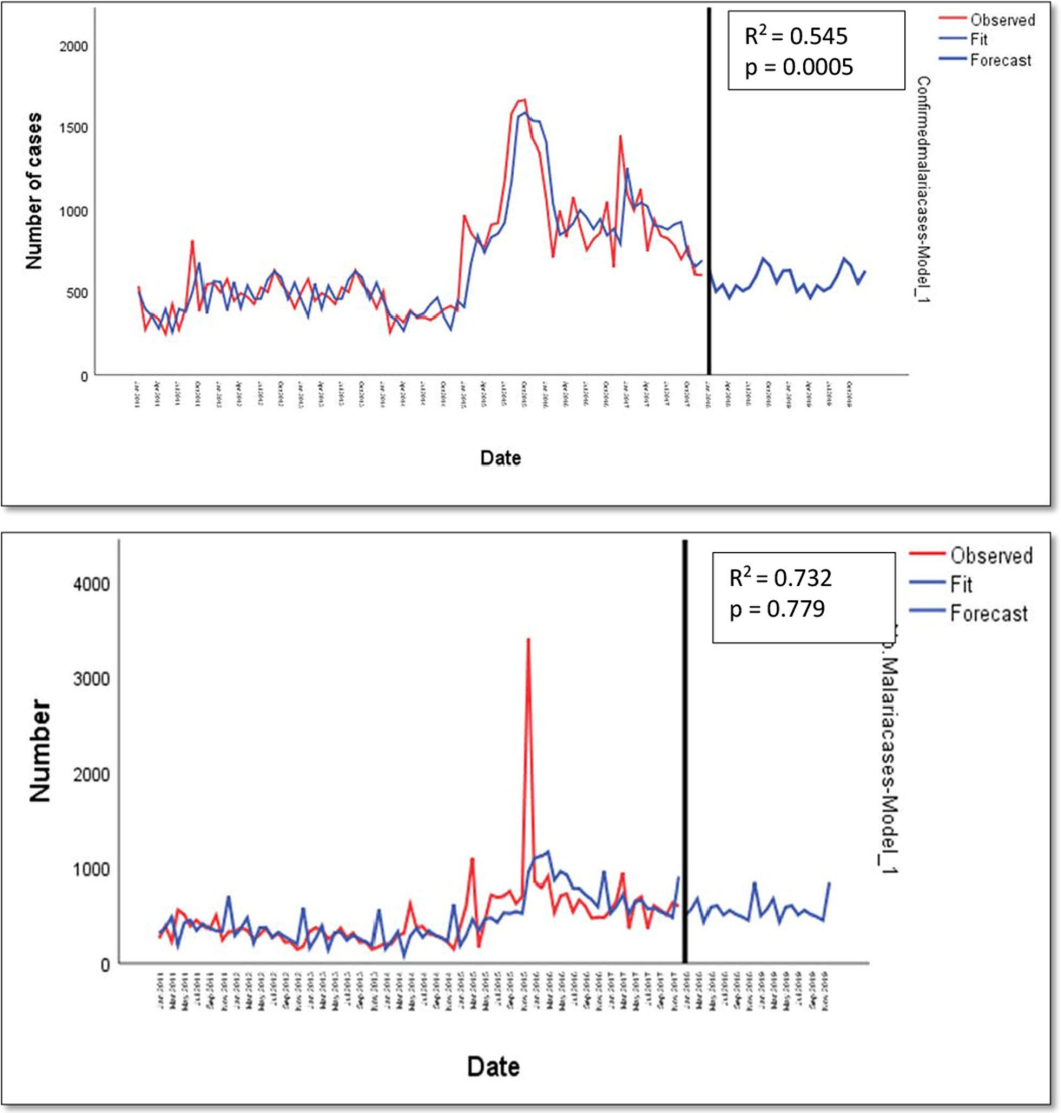
Forecast of number of malaria cases using the simple seasonal model for Muyuka (top) and Tiko (bottom) Health Districts

A two years forecast of the number of malaria cases was done for Muyuka and Tiko Health Districts following their trends over the seven years period under study. The forecast was done using the simple seasonal model. Figure 7 shows the predicted and the observed trend in the number of malaria cases in Muyuka and Tiko Health Districts. The model statistics showed that Tiko had a better fit than Muyuka with a stationary R-squared value of 0.732 as compared to 0.545 in Muyuka. The Ljung which determines the strength of autocorrelation in the data was significant in Muyuka (p = 0.0005) meaning that there was still an autocorrelation in the data whereas it was not significant in Tiko (0.779) meaning that there was no autocorrelation in the data set. In Tiko Health district, 6738 cases was predicted for 2018 and 7327 cases were observed, while 6738 cases were predicted for 2019 and 21,735 were observed. In Muyuka Health District, 6842 malaria cases were predicted to occur in 2018 and 3167 cases were observed while 6842 cases were predicted in 2019 and 2848 cases were observed.

## Discussion

Although the number of malaria cases reported in this study was limited to the population of inhabitants seeking for medical care in hospitals, with no consideration for those who practice auto-medication, the data shows that the incidence of malaria is high in both MHD and THD. The high burden of malaria in Muyuka Health District reported in this study, confirms previous findings where 98.5% of febrile children admitted in Muyuka District Hospital had malaria [17]. The population of MHD and THD have received mass distribution of long-lasting insecticide treated bed nets three times in 2011 [18], 2015 and 2019 simultaneously [19], however discrepancies in the effective usage abound, which can have a toll on the number of malaria cases. Also studies on the spread of resistant alleles of *Anopheles funestus* in Tiko and in Meanja, found in Muyuka, reported similar vector composition (*Anopheles gambiae*, *Anopheles funestus*, *Anopheles coluzzii*, *Anopheles melas*, *Anopheles hancocki*, *Anopheles nili* and *Anopheles ziemanni*) with differences in relative abundance [20] and *Anopheles melas* which is common in coastal areas, does not contribute to malaria transmission in Tiko [21]. The differences in relative abundance may also affect the number of malaria cases. Mean yearly humidity showed a strong negative correlation with number of malaria cases in MHD, (an inland setting). This implies that in an inland area, higher humidity leads to lower number of malaria cases and inland areas are known to suffer from frequent fluctuation in humidity compared to coastal areas which enjoy a fairly stable humidity as a result of constant evaporation of water from the sea [12]. This observation of a negative correlation between humidity and the number of malaria cases was also true for Tiko, a coastal town but was not significant. Probably because the humidity in coastal areas is fairly constant all year round, as a result of the buffering effect of the sea [12]. This is explained by the fact that Humidity is the amount of water vapor in the air and is inversely proportional to temperature. So in an inland setting where there is rapid increase in temperature during the day, the humidity is lower and vice versa at night whereas in the coastal setting temperature increases more slowly due to the fact that the sea absorbs heat at a slower rate than land and also releases heat at night at that same rate, this keeps the humidity of the environment mild and therefore humidity tend not to have so much impact on the number of malaria cases. Similar results of a negative correlation between humidity and malaria incidence have been recorded in a study carried out in Kahnouj (Iran) [22]. Other studies have shown that relative humidity and malaria incidence have a positive correlation [8] while others show that they have no significant correlation [23].

Mean seasonal temperature showed a moderate positive correlation with number of malaria cases in THD, a typical coastal setting. This implies that high temperatures lead to increase number of malaria cases, and similar results have been reported in Bolifamba, which is located in Fako Division, South West Region of Cameroon [24]. It is thought that increase temperature by 1 °C in the range of 18-26 °C increases the life span of a mosquito by more than a week [7], leading to increase number of mosquitos and also shortens the intrinsic incubation period of malaria parasite within the mosquito vector [25], which leads to increased number of infective bites and increased malaria prevalence. However, in Muyuka Health District, a very weak negative correlation was observed between the number of malaria cases and mean seasonal temperature. Other studies have also shown a significant positive correlation between malaria incidence and temperature [22, 26–29].

Mean seasonal rain fall in Tiko Health District also showed a moderately negative correlation with number of malaria cases. This is in agreement with the fact that excess rainfall washes off mosquito breeding sites and reduces malaria prevalence [9]. This was contrary to what was observed in Muyuka Health District, an inland setting where a weak positive correlation was observed between seasonal rainfall and number of malaria case. This can be explained by the fact that coastal areas usually enjoy more rain fall from convectional rainfall, compared to inland areas like Muyuka Health District. Consequently, Muyuka may have less episodes of excessive rain fall, which can wash off the mosquito breeding sites, rather the less rainfall creates new breeding sites by filling up potholes, which explains the positive correlation. Another study has also reported that rainfall has a decreasing effect on *Plasmodium vivax* prevalence [30].

Finally, prediction of future number of malaria cases was done using the simple seasonal model following the trend of malaria incidence for a 7-year period. These predictions were for two years after the study period (2018 and 2019). The observed and fitted values were well fitted (Fig. 7) with a stationary R squared value of 0.545 for MHD and 0.732 for THD. The prediction showed a higher number of malaria cases in 2018 and 2019 (6842 for both years) than the number observes (3167 in 2018 and 2848 in 2019) in MHD. In THD, the model predicted less than what was observed. In THD the model showed that there will be 6738 cases of malaria in 2018 and 2019 as opposed to 7327 cases that was observed in 2018 and 21,735 in 2019. These predictions are informative but far from accuracy as a result of other anthropogenic activities which influences the number of malaria cases, such as large-scale use of long-lasting insecticide bed nets, auto-medication, lots of outdoor activities which carry on late into the night during end of year festivities, amongst other. Future models should consider other parameters that influence the incidence of malaria.

## Conclusions

Humidity is the most important climatic parameter that determines the number of malaria cases in Muyuka Health District, while seasonal temperature and rainfall significantly influences the number of malaria cases in Tiko Health District.

## Supplementary Information

**Additional file 1: Table S1.** Muyuka Health District monthly malaria and climatic data.

**Additional file 2: Table S2.** Tiko Health District monthly malaria and climatic data.

**Additional file 3: Table S3. **Observed and predicted number of malaria cases in Muyuka.

**Additional file 4: Table S4.** Observed and predicted number of malaria cases in Tiko from 2018 to 2019.

## Data Availability

Summaries of the raw data used in this manuscript are available in the Additional files 1, 2, 3 and 4, and the entire data set will be made available to anyone, upon reasonable request channeled to the corresponding author. The Malaria data used in this study is available on the website of the DHIS2 platform of the Ministry of Public Health, Cameroon: https://dhis-minsante-cm.org/dhis-web-commons/security/login.action, which can be accessed with permission from the Regional Delegation of Public health.

## Abbreviations

THD: Tiko health district
MHD: Muyuka health district
DHIS2: District health information system 2
RDPH: Regional delegation of public health
RDT: Rapid diagnostic test
