# Supplementary material for: The effect of climatic factors on the number of malaria cases in an inland and a coastal setting from 2011 to 2017 in the equatorial rain forest of Cameroon
Source: BMC Infect Dis. 2022 May 13;22:461. doi: 10.1186/s12879-022-07445-9 (PMC9101852; doi:10.1186/s12879-022-07445-9)
Supplement: Supplementary file 1 — Additional file 1: Table S1. Muyuka Health District monthly malaria and climatic data. [file 12879_2022_7445_MOESM1_ESM.docx]

## Table S1: Muyuka Health District monthly malaria and climatic data

| **Year** | **Month** | **Estimated population** | **Confirmed malaria cases** | **Incidence per 100** | **Rainfall** | **Temperature** | **RH** |
| --- | --- | --- | --- | --- | --- | --- | --- |
| 2011 | January | 92170 | 534 | 5.79 | 6.4 | 26.00 | 76 |
| 2011 | February | 92170 | 277 | 3.01 | 33.0 | 26.50 | 72 |
| 2011 | March | 92170 | 365 | 3.96 | 40.8 | 26.60 | 75 |
| 2011 | April | 92170 | 337 | 3.66 | 150.9 | 26.60 | 78 |
| 2011 | May | 92170 | 249 | 2.70 | 190.3 | 26.10 | 82 |
| 2011 | June | 92170 | 422 | 4.58 | 298.4 | 25.40 | 88 |
| 2011 | July | 92170 | 275 | 2.98 | 570.0 | 24.20 | 90 |
| 2011 | August | 92170 | 404 | 4.38 | 400.0 | 24.20 | 92 |
| 2011 | September | 92170 | 812 | 8.81 | 380.0 | 24.20 | 93 |
| 2011 | October | 92170 | 383 | 4.16 | 300.0 | 24.80 | 89 |
| 2011 | November | 92170 | 545 | 5.91 | 60.9 | 25.10 | 79 |
| 2011 | December | 92170 | 553 | 6.00 | 16.0 | 25.80 | 79 |
| 2012 | January | 94474 | 496 | 5.25 | 12.4 | 27.70 | 76 |
| 2012 | February | 94474 | 576 | 6.10 | 45.0 | 27.70 | 79 |
| 2012 | March | 94474 | 445 | 4.71 | 30.9 | 28.70 | 79 |
| 2012 | April | 94474 | 491 | 5.20 | 130.8 | 28.70 | 83 |
| 2012 | May | 94474 | 468 | 4.95 | 220.0 | 27.70 | 80 |
| 2012 | June | 94474 | 425 | 4.50 | 300.4 | 26.70 | 85 |
| 2012 | July | 94474 | 526 | 5.57 | 450.2 | 28.70 | 85 |
| 2012 | August | 94474 | 497 | 5.26 | 500.8 | 25.70 | 88 |
| 2012 | September | 94474 | 630 | 6.67 | 399.9 | 26.70 | 78 |
| 2012 | October | 94474 | 546 | 5.78 | 284.4 | 26.70 | 84 |
| 2012 | November | 94474 | 494 | 5.23 | 150.2 | 27.70 | 86 |
| 2012 | December | 94474 | 400 | 4.23 | 2.2 | 27.70 | 80 |
| 2013 | January | 95811 | 496 | 5.18 | 0.0 | 27.70 | 79 |
| 2013 | February | 95811 | 576 | 6.01 | 39.2 | 28.70 | 79 |
| 2013 | March | 95811 | 445 | 4.64 | 50.0 | 27.70 | 81 |
| 2013 | April | 95811 | 491 | 5.12 | 99.0 | 27.70 | 81 |
| 2013 | May | 95811 | 468 | 4.88 | 250.5 | 28.70 | 82 |
| 2013 | June | 95811 | 425 | 4.44 | 211.4 | 25.70 | 86 |
| 2013 | July | 95811 | 526 | 5.49 | 499.9 | 24.70 | 89 |
| 2013 | August | 95811 | 497 | 5.19 | 600.4 | 25.70 | 89 |
| 2013 | September | 95811 | 630 | 6.58 | 250.8 | 26.70 | 87 |
| 2013 | October | 95811 | 546 | 5.70 | 266.6 | 26.70 | 83 |
| 2013 | November | 95811 | 494 | 5.16 | 160.4 | 27.70 | 86 |
| 2013 | December | 95811 | 400 | 4.17 | 8.8 | 27.70 | 84 |
| 2014 | January | 98206 | 501 | 5.10 | 10.0 | 28.70 | 81 |
| 2014 | February | 98206 | 260 | 2.65 | 29.9 | 28.70 | 80 |
| 2014 | March | 98206 | 355 | 3.61 | 39.8 | 27.70 | 82 |
| 2014 | April | 98206 | 318 | 3.24 | 100.0 | 27.70 | 84 |
| 2014 | May | 98206 | 388 | 3.95 | 215.5 | 28.70 | 82 |
| 2014 | June | 98206 | 344 | 3.50 | 259.9 | 25.70 | 85 |
| 2014 | July | 98206 | 349 | 3.55 | 560.6 | 24.70 | 88 |
| 2014 | August | 98206 | 333 | 3.39 | 699.9 | 25.70 | 88 |
| 2014 | September | 98206 | 358 | 3.65 | 350.4 | 26.70 | 88 |
| 2014 | October | 98206 | 394 | 4.01 | 284.0 | 26.70 | 86 |
| 2014 | November | 98206 | 414 | 4.22 | 158.0 | 27.70 | 84 |
| 2014 | December | 98206 | 386 | 3.93 | 20.0 | 27.70 | 82 |
| 2015 | January | 100492 | 966 | 9.61 | 6.5 | 27.70 | 76 |
| 2015 | February | 100492 | 857 | 8.53 | 36.8 | 28.70 | 78 |
| 2015 | March | 100492 | 805 | 8.01 | 47.7 | 27.70 | 80 |
| 2015 | April | 100492 | 766 | 7.62 | 87.6 | 27.70 | 80 |
| 2015 | May | 100492 | 907 | 9.03 | 157.6 | 28.70 | 77 |
| 2015 | June | 100492 | 917 | 9.13 | 240.8 | 26.70 | 83 |
| 2015 | July | 100492 | 1173 | 11.67 | 549.9 | 25.70 | 83 |
| 2015 | August | 100492 | 1577 | 15.69 | 730.1 | 25.70 | 84 |
| 2015 | September | 100492 | 1652 | 16.44 | 234.8 | 25.70 | 83 |
| 2015 | October | 100492 | 1660 | 16.52 | 269.7 | 25.70 | 82 |
| 2015 | November | 100492 | 1425 | 14.18 | 204.3 | 26.70 | 79 |
| 2015 | December | 100492 | 1346 | 13.39 | 2.7 | 27.70 | 78 |
| 2016 | January | 102904 | 1061 | 10.31 | 0.0 | 28.70 | 73 |
| 2016 | February | 102904 | 707 | 6.87 | 0.0 | 29.70 | 76 |
| 2016 | March | 102904 | 992 | 9.64 | 71.6 | 28.70 | 78 |
| 2016 | April | 102904 | 831 | 8.08 | 112.0 | 28.70 | 80 |
| 2016 | May | 102904 | 1076 | 10.46 | 220.5 | 27.70 | 81 |
| 2016 | June | 102904 | 900 | 8.75 | 235.8 | 27.70 | 84 |
| 2016 | July | 102904 | 753 | 7.32 | 272.0 | 26.70 | 84 |
| 2016 | August | 102904 | 819 | 7.96 | 247.1 | 26.70 | 88 |
| 2016 | September | 102904 | 857 | 8.33 | 184.5 | 26.70 | 88 |
| 2016 | October | 102904 | 1048 | 10.18 | 119.4 | 26.70 | 88 |
| 2016 | November | 102904 | 649 | 6.31 | 76.5 | 26.70 | 85 |
| 2016 | December | 102904 | 1444 | 14.03 | 9.9 | 28.70 | 81 |
| 2017 | January | 111017 | 1095 | 9.86 | 4.9 | 27.70 | 75 |
| 2017 | February | 111017 | 994 | 8.95 | 0.0 | 28.70 | 75 |
| 2017 | March | 111017 | 1124 | 10.12 | 209.0 | 28.70 | 75 |
| 2017 | April | 111017 | 746 | 6.72 | 187.9 | 27.70 | 80 |
| 2017 | May | 111017 | 939 | 8.46 | 276.3 | 27.70 | 82 |
| 2017 | June | 111017 | 839 | 7.56 | 234.9 | 27.70 | 83 |
| 2017 | July | 111017 | 824 | 7.42 | 509.0 | 25.70 | 89 |
| 2017 | August | 111017 | 781 | 7.03 | 588.9 | 24.70 | 90 |
| 2017 | September | 111017 | 697 | 6.28 | 251.1 | 25.70 | 86 |
| 2017 | October | 111017 | 772 | 6.95 | 140.7 | 26.70 | 85 |
| 2017 | November | 111017 | 604 | 5.44 | 172.7 | 26.70 | 80 |
| 2017 | December | 111017 | 600 | 5.40 | 0.0 | 28.70 | 76 |

RH: relative humidity
