# Supplementary material for: The effect of climatic factors on the number of malaria cases in an inland and a coastal setting from 2011 to 2017 in the equatorial rain forest of Cameroon
Source: BMC Infect Dis. 2022 May 13;22:461. doi: 10.1186/s12879-022-07445-9 (PMC9101852; doi:10.1186/s12879-022-07445-9)
Supplement: Supplementary file 2 — Additional file 2: Table S2. Tiko Health District monthly malaria and climatic data. [file 12879_2022_7445_MOESM2_ESM.docx]

## Table S2: Tiko Health District monthly malaria and climatic data

| **Year** | **Month** | **Temperature** | **RH** | **Rainfall** | **confirmed Malaria cases** | **Population** | **Incidence per 100** |
| --- | --- | --- | --- | --- | --- | --- | --- |
| 2011 | January | 27.00 | 78.00 | 0.00 | 261 | 131596 | 1.98 |
| 2011 | February | 28.00 | 74.00 | 33.90 | 392 | 131596 | 2.98 |
| 2011 | March | 28.00 | 77.00 | 52.60 | 229 | 131596 | 1.74 |
| 2011 | April | 28.00 | 80.00 | 102.50 | 560 | 131596 | 4.26 |
| 2011 | May | 28.00 | 84.00 | 122.40 | 514 | 131596 | 3.91 |
| 2011 | June | 27.00 | 90.00 | 536.90 | 390 | 131596 | 2.96 |
| 2011 | July | 25.00 | 92.00 | 700.30 | 454 | 131596 | 3.45 |
| 2011 | August | 25.00 | 94.00 | 802.20 | 392 | 131596 | 2.98 |
| 2011 | September | 26.00 | 95.00 | 789.10 | 362 | 131596 | 2.75 |
| 2011 | October | 26.00 | 91.00 | 629.70 | 503 | 131596 | 3.82 |
| 2011 | November | 27.00 | 81.00 | 24.10 | 245 | 131596 | 1.86 |
| 2011 | December | 28.00 | 81.00 | 0.00 | 332 | 131596 | 2.52 |
| 2012 | January | 28.00 | 78.00 | 0.00 | 335 | 134886 | 2.48 |
| 2012 | February | 28.00 | 81.00 | 62.30 | 373 | 134886 | 2.77 |
| 2012 | March | 29.00 | 81.00 | 29.30 | 346 | 134886 | 2.57 |
| 2012 | April | 29.00 | 85.00 | 140.10 | 256 | 134886 | 1.90 |
| 2012 | May | 28.00 | 82.00 | 140.60 | 299 | 134886 | 2.22 |
| 2012 | June | 27.00 | 87.00 | 423.40 | 370 | 134886 | 2.74 |
| 2012 | July | 29.00 | 87.00 | 732.00 | 266 | 134886 | 1.97 |
| 2012 | August | 26.00 | 90.00 | 172.60 | 319 | 134886 | 2.36 |
| 2012 | September | 27.00 | 80.00 | 209.20 | 221 | 134886 | 1.64 |
| 2012 | October | 27.00 | 86.00 | 224.80 | 226 | 134886 | 1.68 |
| 2012 | November | 28.00 | 86.00 | 87.40 | 146 | 134886 | 1.08 |
| 2012 | December | 28.00 | 82.00 | 0.00 | 176 | 134886 | 1.30 |
| 2013 | January | 28.00 | 81.00 | 48.00 | 335 | 136897 | 2.45 |
| 2013 | February | 29.00 | 81.00 | 41.00 | 373 | 136897 | 2.72 |
| 2013 | March | 28.00 | 83.00 | 85.30 | 346 | 136897 | 2.53 |
| 2013 | April | 28.00 | 83.00 | 186.30 | 256 | 136897 | 1.87 |
| 2013 | May | 29.00 | 84.00 | 239.40 | 299 | 136897 | 2.18 |
| 2013 | June | 26.00 | 88.00 | 609.40 | 370 | 136897 | 2.70 |
| 2013 | July | 25.00 | 91.00 | 571.10 | 266 | 136897 | 1.94 |
| 2013 | August | 26.00 | 91.00 | 682.40 | 319 | 136897 | 2.33 |
| 2013 | September | 27.00 | 89.00 | 288.70 | 221 | 136897 | 1.61 |
| 2013 | October | 27.00 | 85.00 | 239.30 | 226 | 136897 | 1.65 |
| 2013 | November | 28.00 | 88.00 | 82.70 | 146 | 136897 | 1.07 |
| 2013 | December | 28.00 | 86.00 | 67.40 | 176 | 136897 | 1.29 |
| 2014 | January | 29.00 | 83.00 | 0.00 | 208 | 140319 | 1.48 |
| 2014 | February | 29.00 | 82.00 | 0.00 | 203 | 140319 | 1.45 |
| 2014 | March | 28.00 | 84.00 | 119.10 | 293 | 140319 | 2.09 |
| 2014 | April | 28.00 | 86.00 | 191.70 | 315 | 140319 | 2.24 |
| 2014 | May | 29.00 | 84.00 | 127.30 | 622 | 140319 | 4.43 |
| 2014 | June | 26.00 | 87.00 | 185.30 | 374 | 140319 | 2.67 |
| 2014 | July | 25.00 | 90.00 | 458.30 | 387 | 140319 | 2.76 |
| 2014 | August | 26.00 | 90.00 | 406.10 | 308 | 140319 | 2.19 |
| 2014 | September | 27.00 | 90.00 | 218.90 | 293 | 140319 | 2.09 |
| 2014 | October | 27.00 | 88.00 | 157.80 | 269 | 140319 | 1.92 |
| 2014 | November | 28.00 | 86.00 | 128.90 | 222 | 140319 | 1.58 |
| 2014 | December | 28.00 | 84.00 | 0.00 | 149 | 140319 | 1.06 |
| 2015 | January | 28.00 | 78.00 | 0.00 | 373 | 143586 | 2.60 |
| 2015 | February | 29.00 | 80.00 | 21.10 | 603 | 143586 | 4.20 |
| 2015 | March | 28.00 | 82.00 | 38.70 | 1096 | 143586 | 7.63 |
| 2015 | April | 28.00 | 82.00 | 45.30 | 169 | 143586 | 1.18 |
| 2015 | May | 29.00 | 79.00 | 98.00 | 449 | 143586 | 3.13 |
| 2015 | June | 27.00 | 85.00 | 382.40 | 719 | 143586 | 5.01 |
| 2015 | July | 26.00 | 85.00 | 384.80 | 692 | 143586 | 4.82 |
| 2015 | August | 26.00 | 86.00 | 546.20 | 704 | 143586 | 4.90 |
| 2015 | September | 26.00 | 85.00 | 215.40 | 759 | 143586 | 5.29 |
| 2015 | October | 26.00 | 84.00 | 275.10 | 627 | 143586 | 4.37 |
| 2015 | November | 27.00 | 81.00 | 131.90 | 699 | 143586 | 4.87 |
| 2015 | December | 28.00 | 80.00 | 4.60 | 3398 | 143586 | 23.67 |
| 2016 | January | 29.00 | 75.00 | 0.00 | 851 | 147032 | 5.79 |
| 2016 | February | 30.00 | 78.00 | 0.00 | 797 | 147032 | 5.42 |
| 2016 | March | 29.00 | 80.00 | 71.60 | 906 | 147032 | 6.16 |
| 2016 | April | 29.00 | 82.00 | 112.00 | 531 | 147032 | 3.61 |
| 2016 | May | 28.00 | 83.00 | 220.50 | 709 | 147032 | 4.82 |
| 2016 | June | 28.00 | 86.00 | 235.80 | 730 | 147032 | 4.96 |
| 2016 | July | 27.00 | 86.00 | 272.00 | 542 | 147032 | 3.69 |
| 2016 | August | 27.00 | 90.00 | 247.10 | 667 | 147032 | 4.54 |
| 2016 | September | 27.00 | 90.00 | 184.50 | 599 | 147032 | 4.07 |
| 2016 | October | 27.00 | 90.00 | 119.40 | 475 | 147032 | 3.23 |
| 2016 | November | 27.00 | 87.00 | 76.50 | 484 | 147032 | 3.29 |
| 2016 | December | 29.00 | 83.00 | 9.90 | 481 | 147032 | 3.27 |
| 2017 | January | 28.00 | 77.00 | 4.90 | 553 | 148101 | 3.73 |
| 2017 | February | 29.00 | 77.00 | 0.00 | 659 | 148101 | 4.45 |
| 2017 | March | 29.00 | 77.00 | 209.00 | 945 | 148101 | 6.38 |
| 2017 | April | 28.00 | 82.00 | 187.90 | 367 | 148101 | 2.48 |
| 2017 | May | 28.00 | 84.00 | 276.30 | 655 | 148101 | 4.42 |
| 2017 | June | 28.00 | 85.00 | 234.90 | 700 | 148101 | 4.73 |
| 2017 | July | 26.00 | 91.00 | 509.00 | 366 | 148101 | 2.47 |
| 2017 | August | 25.00 | 92.00 | 588.90 | 607 | 148101 | 4.10 |
| 2017 | September | 26.00 | 88.00 | 251.10 | 562 | 148101 | 3.79 |
| 2017 | October | 27.00 | 87.00 | 140.70 | 508 | 148101 | 3.43 |
| 2017 | November | 27.00 | 82.00 | 172.70 | 635 | 148101 | 4.29 |
| 2017 | December | 29.00 | 78.00 | 0.00 | 600 | 148101 | 4.05 |

RH: Relative humidity
