# Supplementary material for: The effect of climatic factors on the number of malaria cases in an inland and a coastal setting from 2011 to 2017 in the equatorial rain forest of Cameroon
Source: BMC Infect Dis. 2022 May 13;22:461. doi: 10.1186/s12879-022-07445-9 (PMC9101852; doi:10.1186/s12879-022-07445-9)
Supplement: Supplementary file 3 — Additional file 3: Table S3. Observed and predicted number of malaria cases in Muyuka. [file 12879_2022_7445_MOESM3_ESM.docx]

## Table S3: Observed and predicted number of malaria cases in Muyuka

| **Months** | **Actual number of cases** | **Predicted Number of cases** |
| --- | --- | --- |
| January 2018 | 676 | 630 |
| February 2018 | 473 | 501 |
| March 2018 | 433 | 542 |
| April 2018 | 415 | 463 |
| May 2018 | 337 | 537 |
| June 2018 | 192 | 505 |
| July 2018 | 93 | 527 |
| August 2018 | 185 | 596 |
| September 2018 | 73 | 700 |
| October 2018 | 31 | 659 |
| November 2018 | 109 | 555 |
| December 2018 |  | 627 |
| **Total** | **3167** | **6842** |
| January 2019 | 215 | 630 |
| February 2019 | 135 | 501 |
| March 2019 | 141 | 542 |
| April 2019 | 214 | 463 |
| May 2019 | 293 | 537 |
| June 2019 | 313 | 505 |
| July 2019 | 315 | 527 |
| August 2019 | 243 | 596 |
| September 2019 | 140 | 700 |
| October 2019 | 189 | 659 |
| November 2019 | 343 | 555 |
| December 2019 | 307 | 627 |
| **Total** | **2848** | **6842** |
| Stationary R squared value | 0.545 | |
| Ljung Box significance | 0.0005 | |
