# Supplementary material for: The effect of climatic factors on the number of malaria cases in an inland and a coastal setting from 2011 to 2017 in the equatorial rain forest of Cameroon
Source: BMC Infect Dis. 2022 May 13;22:461. doi: 10.1186/s12879-022-07445-9 (PMC9101852; doi:10.1186/s12879-022-07445-9)
Supplement: Supplementary file 4 — Additional file 4: Table S4. Observed and predicted number of malaria cases in Tiko from 2018 to 2019. [file 12879_2022_7445_MOESM4_ESM.docx]

## Table S4: Observed and predicted number of malaria cases in Tiko from 2018 to 2019

| **Month/year** | **Observed number of cases** | **Predicted number of cases** |
| --- | --- | --- |
| January 2018 | 258 | 500 |
| February 2018 | 516 | 569 |
| March 2018 | 1008 | 678 |
| April 2018 | 830 | 434 |
| May 2018 | 735 | 590 |
| June 2018 | 622 | 605 |
| July 2018 | 466 | 508 |
| August 2018 | 595 | 557 |
| September 2018 | 419 | 515 |
| October 2018 | 116 | 488 |
| November 2018 | 868 | 452 |
| December 2018 | 894 | 842 |
| **Total** | **7327** | **6738** |
| January 2019 | 1888 | 500 |
| February 2019 | 1703 | 569 |
| March 2019 | 1655 | 678 |
| April 2019 | 2029 | 434 |
| May 2019 | 2101 | 590 |
| June 2019 | 1931 | 605 |
| July 2019 | 2274 | 508 |
| August 2019 | 2129 | 557 |
| September 2019 | 1601 | 515 |
| October 2019 | 1409 | 488 |
| November 2019 | 1566 | 452 |
| December 2019 | 1449 | 842 |
| **Total** | **21735** | **6738** |
| Stationary R squared | 0.732 | |
| Ljung Box Q Significance | 0.779 | |
